# Heat-Killed *Ligilactobacillus salivarius* FFIG56 Modulates Innate Immune Response in Bovine Mammary Epithelial Cells

**DOI:** 10.3390/ijms27219809

**Published:** 2026-11-01

**Authors:** Kohtaro Fukuyama, Leonardo Albarracin, Luciano Arellano-Arriagada, Weichen Gong, Keita Nishiyama, Yoshinobu Uemoto, Satoshi Haga, Hitoshi Shirakawa, Hisashi Aso, Julio Villena, Haruki Kitazawa

**Affiliations:** 1 Food and Feed Immunology Group, Laboratory of Animal Food Function, Graduate School of Agricultural Science, Tohoku University, Sendai 980-8572, Miyagi, Japan; 2 Livestock Immunology Unit, International Education and Research Centre for Food and Agricultural Immunology (CFAI), Graduate School of Agricultural Science, Tohoku University, Sendai 980-8572, Miyagi, Japan; 3 Laboratory of Respiratory Immunology (LaRI), Division of Animal Immunology and Omics, International Education and Research Center for Food and Agricultural Immunology (CFAI), Graduate School of Agricultural Science, Tohoku University, Sendai 980-8572, Miyagi, Japan; 4 Laboratory of Animal Breeding and Genetics, Graduate School of Agricultural Science, Tohoku University, Sendai 980-8572, Miyagi, Japan; 5 Laboratory of Animal Physiology, Graduate School of Agricultural Science, Tohoku University, Sendai 980-8572, Miyagi, Japan; 6 Laboratory of Nutrition, Graduate School of Agricultural Science, Tohoku University, Sendai 980-8572, Miyagi, Japan; 7 Food and Health Evaluation Unit, International Education and Research Centre for Food and Agricultural Immunology (CFAI), Graduate School of Agricultural Science, Tohoku University, Sendai 980-8572, Miyagi, Japan; 8 Laboratory of Animal Health Science, Graduate School of Agricultural Science, Tohoku University, Sendai 980-8572, Miyagi, Japan; 9 The Cattle Museum, Oshu 029-4205, Iwate, Japan

**Keywords:** *Ligilactobacillus salivarius* FFIG56, bovine mammary epithelial, TLR4, inflammation, immunobiotics, postimmunobiotics

## Abstract

Immunomodulatory lactic acid bacteria (immunobiotics) have been proposed to prevent bovine mastitis through the enhancement of host immune responses. Furthermore, accumulating evidence suggests that non-viable immunobiotics (postimmunobiotics) can be as effective as viable bacteria in modulating immunity in the bovine host. However, the molecular mechanisms underlying the immunomodulatory effects of heat-inactivated lactic acid bacteria in bovine mammary epithelial cells remain incompletely understood. In the present study, we evaluated the effects of heat-killed *Ligilactobacillus salivarius* FFIG56 (hk-FFIG56) on the Toll-like receptor (TLR)-4-mediated innate immune response in bovine mammary epithelial cells (BMECs) in vitro. BMECs were pre-stimulated with hk-FFIG56 and subsequently challenged with *Escherichia coli*-derived lipopolysaccharide (LPS). Transcriptomic analyses revealed that, compared with cells stimulated with LPS alone, hk-FFIG56-pretreated BMECs exhibited 743 upregulated and 881 downregulated genes, indicating substantial changes in the LPS-induced transcriptional response. Among the inflammatory mediators evaluated, hk-FFIG56 pretreatment significantly reduced LPS-induced interleukin-6 (*IL-6*) and interleukin-8 (*IL-8*) expression at 12 h, as well as *CCL2* expression at 6 and 12 h, compared with cells challenged with LPS alone at the corresponding time points. Differential regulation of the negative regulators of TLR signaling, IRAK-M, Tollip, and Mitogen-activated protein kinase phosphatase 1 (MKP-1), was also detected in BMECs pretreated with hk-FFIG56. In addition, hk-FFIG56 pretreatment prevented LPS-induced reduction in IκBα protein levels, whereas no significant changes were detected in the p-ERK/ERK ratio at the evaluated time points. Furthermore, hk-FFIG56 pretreatment attenuated the LPS-induced reduction in *CSN3* (κ-casein) mRNA expression in BMECs. Overall, these results suggest that hk-FFIG56 modulates LPS-induced inflammatory responses and *CSN3* mRNA expression in BMECs, supporting its potential as a postimmunobiotic candidate for further investigation in the context of bovine mastitis prevention.

## 1. Introduction

Bovine mastitis is among the most prevalent diseases affecting dairy cattle worldwide and represents a major concern for animal health, welfare, and dairy production due to the substantial economic losses it generates. A wide range of microorganisms, including bacteria, viruses, and fungi, are capable of infecting the mammary gland and triggering inflammatory processes [1]. However, bacteria are recognized as the predominant etiological agents of mastitis [2]. Among them, *Escherichia coli* has been consistently identified as one of the leading causes of bacterial mastitis. The pathogenicity of *E. coli* is mediated by multiple virulence factors, such as adhesins, toxins, invasins, hemolysins, and lipopolysaccharide (LPS), which contribute to epithelial damage and the initiation of inflammatory responses in the bovine mammary gland [3]. Notably, LPS is considered the primary virulence determinant, acting as a microbe-associated molecular pattern (MAMP) that activates innate immune signaling through Toll-like receptor 4 (TLR4), thereby promoting inflammatory responses associated with mastitis development [4].

Antibiotics remain an important component of bovine mastitis treatment and control. However, treatment outcomes vary depending on factors such as the causative pathogen, infection severity, and disease chronicity [5,6]. Moreover, although antimicrobial therapy targets bacterial infections, the resolution of infection does not necessarily imply the complete reversal of inflammation-associated mammary tissue damage or the restoration of milk production. The extensive and sometimes prophylactic use of antibiotics in dairy production has also raised significant concerns. One of the most important issues is the selection and spread of antimicrobial-resistant pathogens, which represent a growing threat to both animal and public health. Furthermore, antibiotic use in lactating cows can lead to the presence of drug residues in milk, requiring withdrawal periods that result in economic losses for dairy producers and increasing regulatory pressure to reduce antibiotic usage [7]. In this context, the development of alternative or complementary strategies that enhance the host’s natural defense mechanisms has become an important goal for improving mastitis control in sustainable dairy production systems.

Probiotic lactic acid bacteria (LAB) have been proposed as a promising biotechnological strategy for the prevention and treatment of bovine mastitis. Among probiotics, microorganisms that exert beneficial effects on the host through the modulation of immune responses are referred to as immunobiotics. These immunomodulatory properties are strain-specific and may involve the enhancement of protective immune mechanisms, the regulation of inflammatory responses, or both, depending on the immunological context. It was shown that the intramammary administration of the probiotic *Lactiplantibacillus plantarum* CM49 in mastitic cows modulated the milk microbiological profile, reducing mastitis-associated pathogens while increasing lactobacilli counts, thereby promoting a beneficial shift in the mammary microbial community [8]. Intramammary infusion of live *Lactococcus lactis* DPC3147 in dairy cows with mastitis triggered a strong local innate immune response in the mammary gland, characterized by rapid recruitment of immune cells and increased inflammatory mediators, indicating that LAB can stimulate host defenses against intramammary infections [9]. Similarly, intramammary inoculation of *Schleiferilactobacillus perolens* CRL1724 in lactating cows demonstrated that the strain could persist in the mammary gland without causing tissue damage while inducing a transient immune response [10,11]. Furthermore, studies involving specific LAB strains have demonstrated that certain non-viable immunobiotic preparations (postimmunobiotics) can retain immunomodulatory activities comparable to those of their viable counterparts. In a study involving cows with chronic mastitis, emulsion-based formulations containing either viable or heat-killed *Lactococcus lactis* DPC3147 were administered intramammarily to evaluate their therapeutic effects. Both treatments induced comparable local immune responses, measured by increased interleukin (IL)-8 levels in milk, and showed similar cure outcomes based on reductions in somatic cell counts and pathogen clearance, indicating that heat-inactivated bacteria can be as effective as live cells while offering potential advantages in formulation stability and shelf life [12]. Nevertheless, the immunomodulatory efficacy of heat-inactivated bacteria cannot be generalized across different LAB strains or experimental conditions. The effects of non-viable preparations depend on strain-specific characteristics, the inactivation procedure, and the resulting composition and structural integrity of bacterial components. Furthermore, viable and heat-inactivated bacteria may interact differently with host cells, as bacterial viability influences metabolic activity, the production and release of bioactive molecules, and the availability of microbial structures involved in host recognition. Consequently, although certain heat-killed LAB (hk-LAB) preparations have demonstrated immunomodulatory activities comparable to those of their viable counterparts, such equivalence should be established individually for each strain and experimental context. These considerations highlight the importance of characterizing the immunomodulatory properties of specific hk-LAB preparations rather than extrapolating findings from viable bacteria or other strains.

The mammary epithelium plays an important role in the innate immune response to bacterial infection [13]. Recognition of LPS through TLR4 activates intracellular signaling pathways, including NF-κB and MAPKs, which regulate the expression of inflammatory cytokines and chemokines involved in host defense. These responses are also controlled by negative regulators of TLR signaling that contribute to maintaining immune homeostasis. Therefore, pretreatment of mammary epithelial cells with hk-LAB followed by LPS stimulation provides an experimental approach to investigate whether bacterial preparations can modify subsequent inflammatory responses. The simultaneous evaluation of inflammatory mediators, negative regulators of TLR signaling, and NF-κB/MAPK-associated signaling allows for the characterization of different aspects of this immunomodulatory activity. In addition, because inflammation can affect the physiological functions of mammary epithelial cells [14,15], the expression of milk-protein-related genes, such as κ-casein, may provide complementary information about the influence of hk-LAB on epithelial responses under inflammatory conditions.

Despite previous studies demonstrating the immunomodulatory potential of viable and hk-LAB in the bovine mammary gland, important questions remain regarding the strain-specific effects of non-viable preparations on mammary epithelial innate immunity. In particular, the extent to which hk-LAB modify the global transcriptional response of bovine mammary epithelial cells (BMECs) to LPS stimulation, differentially regulate inflammatory mediators and negative regulators of TLR signaling, and influence NF-κB- and MAPK-associated signaling remains to be further characterized. Therefore, the present study aimed to identify hk-LAB strains capable of modulating LPS-induced inflammatory responses in BMECs and to characterize the immunomodulatory activity of the selected strain, *Ligilactobacillus salivarius* FFIG56. To this end, we evaluated transcriptomic changes and the expression of inflammatory mediators and TLR signaling regulators and selected components of intracellular signaling pathways following hk-FFIG56 pretreatment and subsequent LPS challenge.

## 2. Results

### 2.1. Selection of Heat-Killed Lactobacilli with the Capacity to Diminish LPS-Induced Inflammation in BMECs

**Figure 1:**
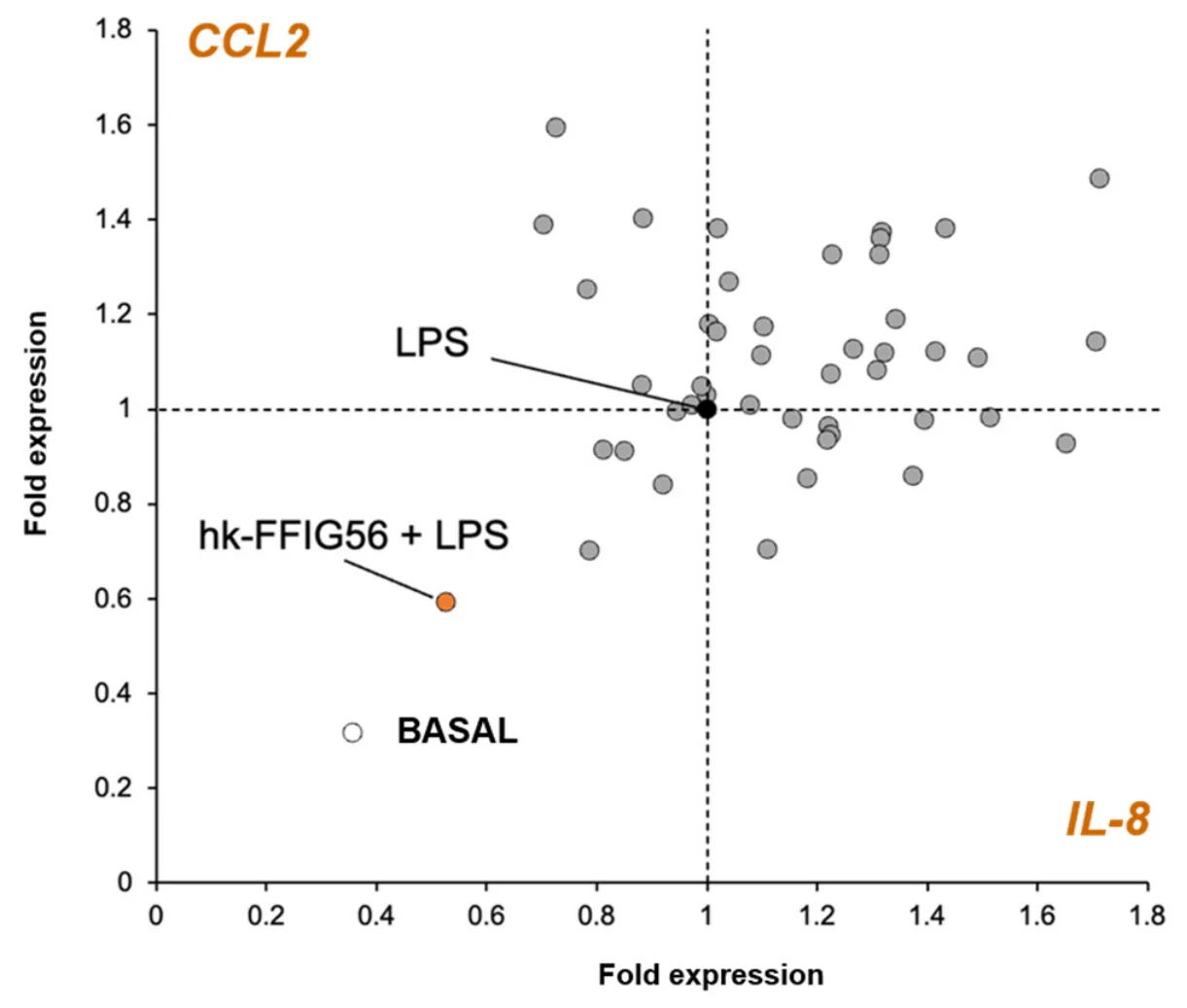
Screening of heat-killed lactic acid bacteria (hk-LAB) for their ability to modulate LPS-induced inflammatory responses in bovine mammary epithelial cells (BMECs). BMECs were pre-stimulated with 46 different hk-LAB strains and subsequently challenged with LPS. The expression levels of *IL-8* (*CXCL8*) and *CCL2* (*MCP-1*) were determined by RT-qPCR. The two-dimensional scatter plot shows the relative expression of *IL-8* and *CCL2* following LPS stimulation in hk-LAB-treated BMECs. Heat-killed *Ligilactobacillus salivarius* FFIG56 (hk-FFIG56) was selected based on its ability to reduce the LPS-induced expression of both inflammatory chemokines.

We first aimed to select hk-LAB capable of modulating LPS-induced inflammation in BMECs. For this purpose, BMECs were pre-stimulated with hk-LAB and challenged with LPS, and the expression of the *IL-8* (or *CXCL8*) and *CCL2* (or *MCP-1*) genes was measured using RT-qPCR (Figure 1). We selected these two genes as biomarkers considering our previous report evaluating the transcriptomic response of BMECs to LPS stimulation that indicated significant upregulation of these two chemokines [16]. This initial evaluation was intended to identify candidate strains for subsequent, more comprehensive characterization of their immunomodulatory properties. As observed in the two-dimensional scatter plot that shows the changes in the expression of *IL-8* and *CCL2*, the different hk-LAB exerted distinct effects, highlighting the strain specificity in the ability to modulate these inflammatory markers in BMECs. Among the 46 strains evaluated, heat-killed *Ligilactobacillus salivarius* FFIG56 (hk-FFIG56) was selected for further characterization based on its ability to reduce the expression of both chemokines following LPS stimulation (Figure 1).

### 2.2. Reduction in LPS-Induced Inflammation in BMECs by hk-FFIG56

To further evaluate the influence of hk-FFIG56 on the transcriptome response of BMECs, RNA sequencing analysis was performed. In these experiments, BMECs were treated with hk-FFIG56 and then challenged with LPS. BMECs treated only with LPS or hk-FFIG56 were used for comparisons. For the Venn diagram analysis, differentially expressed genes were identified separately for each treatment group (LPS, hk-FFIG56, and hk-FFIG56+LPS) relative to untreated control cells. Shared genes were defined as those significantly upregulated or downregulated in more than one treatment group, whereas unique genes were those differentially expressed exclusively in a single treatment group.

**Figure 2:**
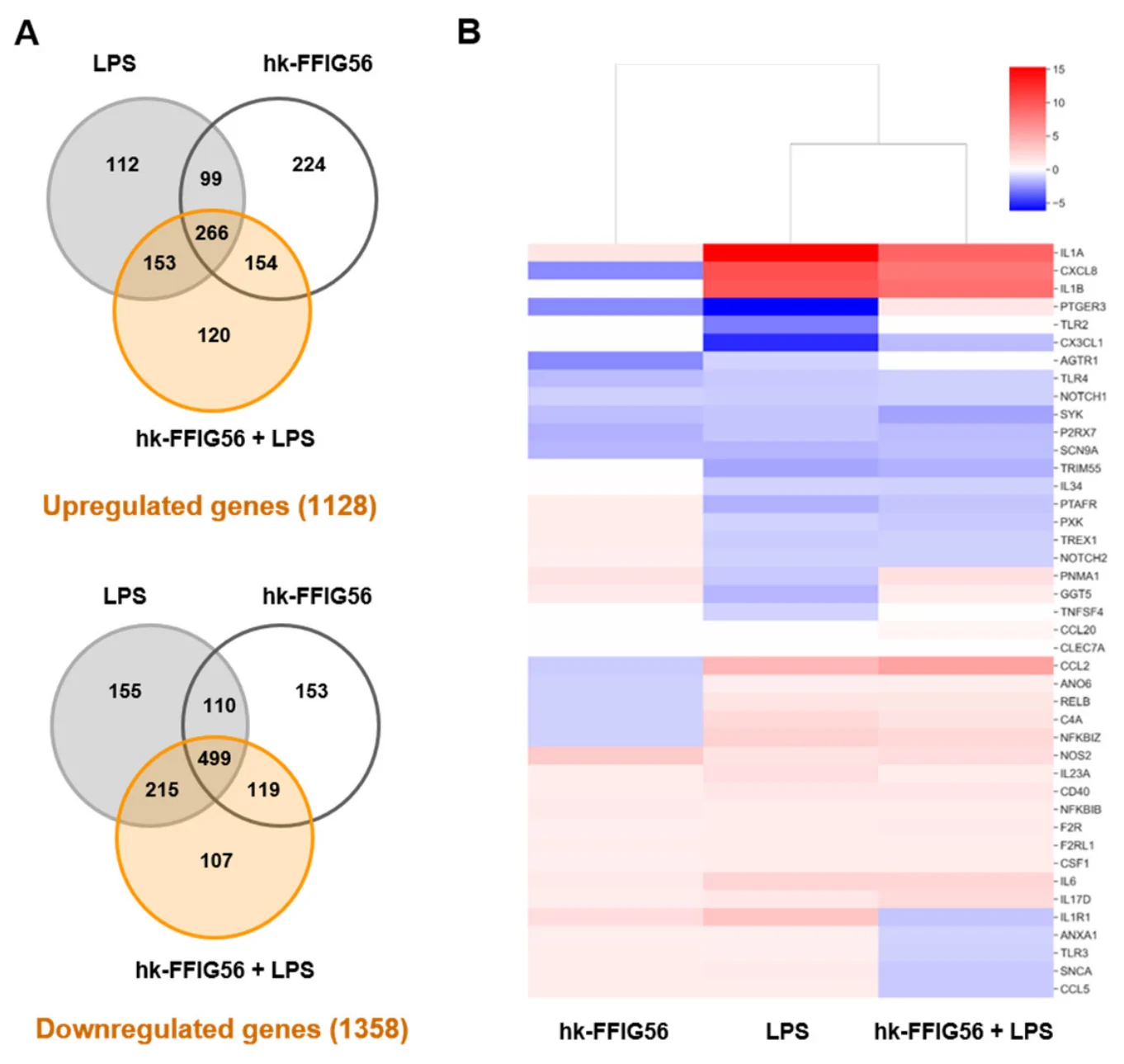
Transcriptomic response of bovine mammary epithelial cells (BMECs) treated with heat-killed *Ligilactobacillus salivarius* FFIG56 (hk-FFIG56) and challenged with LPS. BMECs were pre-stimulated with hk-FFIG56 and subsequently challenged with LPS. BMECs treated only with LPS or hk-FFIG56 were used for comparison. Transcriptomic changes were evaluated by RNA sequencing analysis. (**A**) Venn diagrams showing the distribution and overlap of significantly upregulated and downregulated genes among the LPS, hk-FFIG56, and hk-FFIG56+LPS groups, each compared with untreated control cells. The total numbers of 1128 upregulated and 1358 downregulated genes represent the union of the corresponding gene sets across the three treatment groups. (**B**) Heatmap showing the expression profiles of differentially expressed immune-related genes among the experimental groups.

Across the three experimental treatments (LPS, hk-FFIG56, and hk-FFIG56+LPS), transcriptomic analysis identified a total of 1128 upregulated and 1358 downregulated genes relative to the untreated control group (Supplementary Table S1). In the direct comparison between hk-FFIG56+LPS and LPS-treated cells, 743 genes were upregulated, and 881 were downregulated, highlighting the impact of hk-FFIG56 pretreatment on the LPS-induced transcriptional response. As shown in the Venn diagram analysis (Figure 2A), the three treatments, LPS, hk-FFIG56 and hk-FFIG56+LPS, shared 266 and 499 significantly upregulated and downregulated genes, respectively. Interestingly, hk-FFIG56 treatment induced the upregulation of 224 unique genes, while in the hk-FFIG56+LPS group, there were 120 unique upregulated genes. Similarly, hk-FFIG56 treatment induced the downregulation of 153 unique genes, while in the hk-FFIG56+LPS group, there were 107 unique downregulated genes. Of these differentially regulated genes, several of them were assigned to immune-related functions according to the GO database (Supplementary Table S1).

When the expression of immune-related genes was compared between the groups using heatmap analysis, a clear difference was observed between BMECs treated with hk-FFIG56 and those stimulated with LPS, with or without pretreatment with heat-killed lactobacilli (Figure 2B). In addition, a different transcriptomic response was detected between the LPS and hk-FFIG56+LPS groups. Although LPS stimulation induced increases in the expression of several inflammatory genes in both control and hk-FFIG56-treated BMECs, those treated with the heat-killed lactobacilli had significantly lower levels of inflammatory factors compared with controls. In fact, comparison of fold-changes between the LPS and hk-FFIG56+LPS groups showed differences in *IL-1α* (15.3 vs. 9.1), *IL-1β* (10.1 vs. 8.5), *CXCL8* (10.4 vs. 8.1), *CR1* (7.7 vs. 2.1), *CCL2* (5.5 vs. 4.2), *IL-6* (2.5 vs. 1.4), and *C4A* (2.3 vs. 1.6), among others.

**Figure 3:**
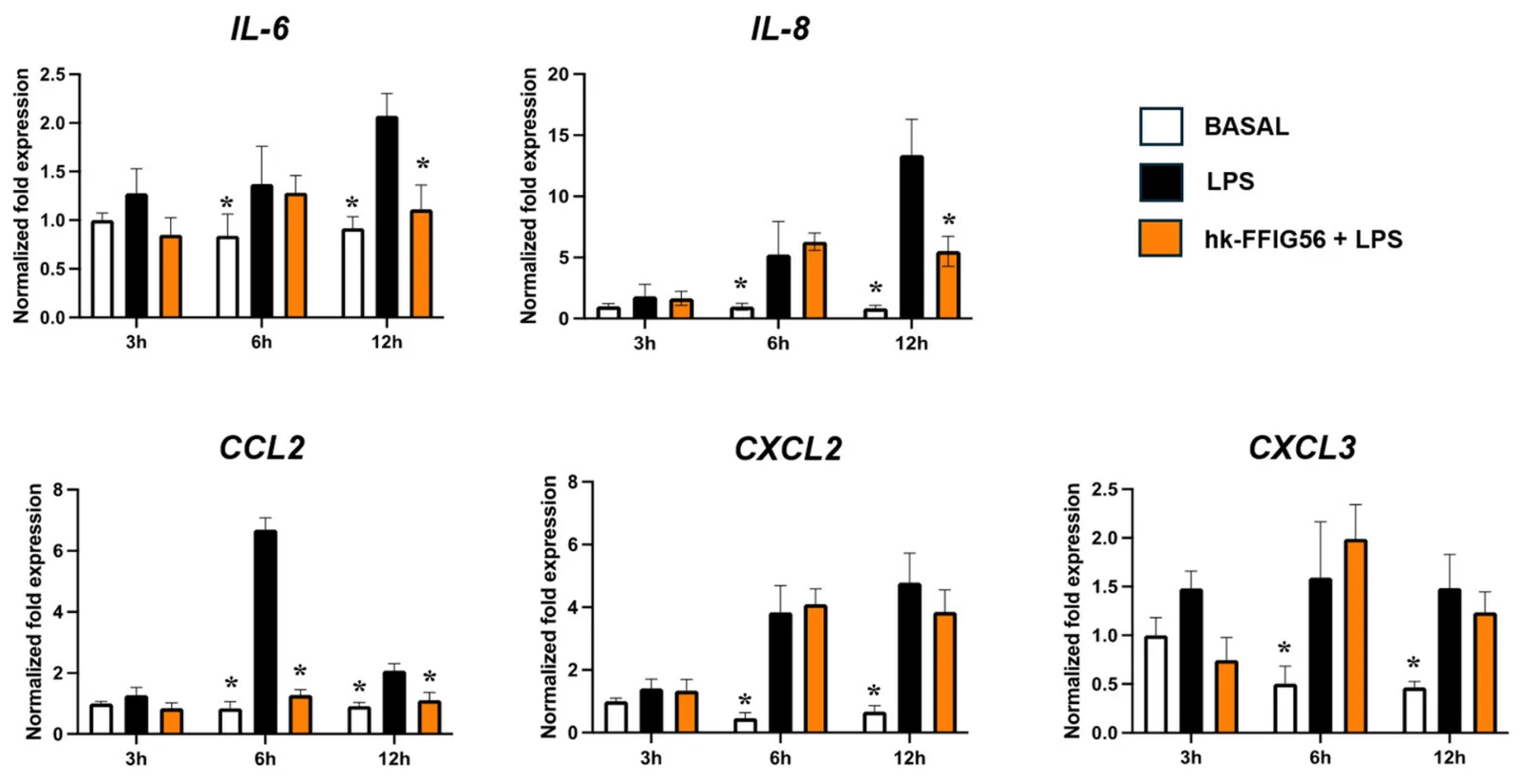
Effect of heat-killed *Ligilactobacillus salivarius* FFIG56 (hk-FFIG56) on LPS-induced inflammatory responses in bovine mammary epithelial cells (BMECs). BMECs were pre-stimulated with hk-FFIG56 and subsequently challenged with LPS. The expression levels of *IL-6*, *IL-8* (*CXCL8*), *CCL2* (*MCP-1*), *CXCL2*, and *CXCL3* were determined by RT-qPCR at 3, 6, and 12 h after LPS stimulation. BMECs stimulated with LPS without hk-FFIG56 pretreatment were used for comparison. The results are shown as mean ± SD (*n* = 3 biological replicates per experimental condition). Significant differences are shown compared to the LPS-stimulated control group at the same time point, *p* < 0.05 (*).

We confirmed the ability of hk-FFIG56 to modulate the response of BMECs to LPS stimulation by performing qPCR with selected genes after 3, 6, and 12 h. As shown in Figure 3, the stimulation of BMECs with LPS increased the expression of *IL-6*, *IL-8*, *CCL2*, *CXCL2*, and *CXCL3* compared to basal levels. The highest expression of *IL-6*, *IL-8* and *CXCL2* was detected at hour 12 post-LPS stimulation, while the peaks of *CCL2* and *CXCL3* were detected at hour 6. Interestingly, the pretreatment of BMECs with hk-FFIG56 significantly reduced the expression of *IL-6* and *IL-8* at hour 12 and *CCL2* at hours 6 and 12. In addition, no significant differences in *CXCL2* or *CXCL3* mRNA expression were detected between LPS-stimulated BMECs with and without hk-FFIG56 pretreatment at any of the time points evaluated (Figure 3).

### 2.3. Modulation of Negative Regulators of TLR Signaling in BMECs by hk-FFIG56

**Figure 4:**
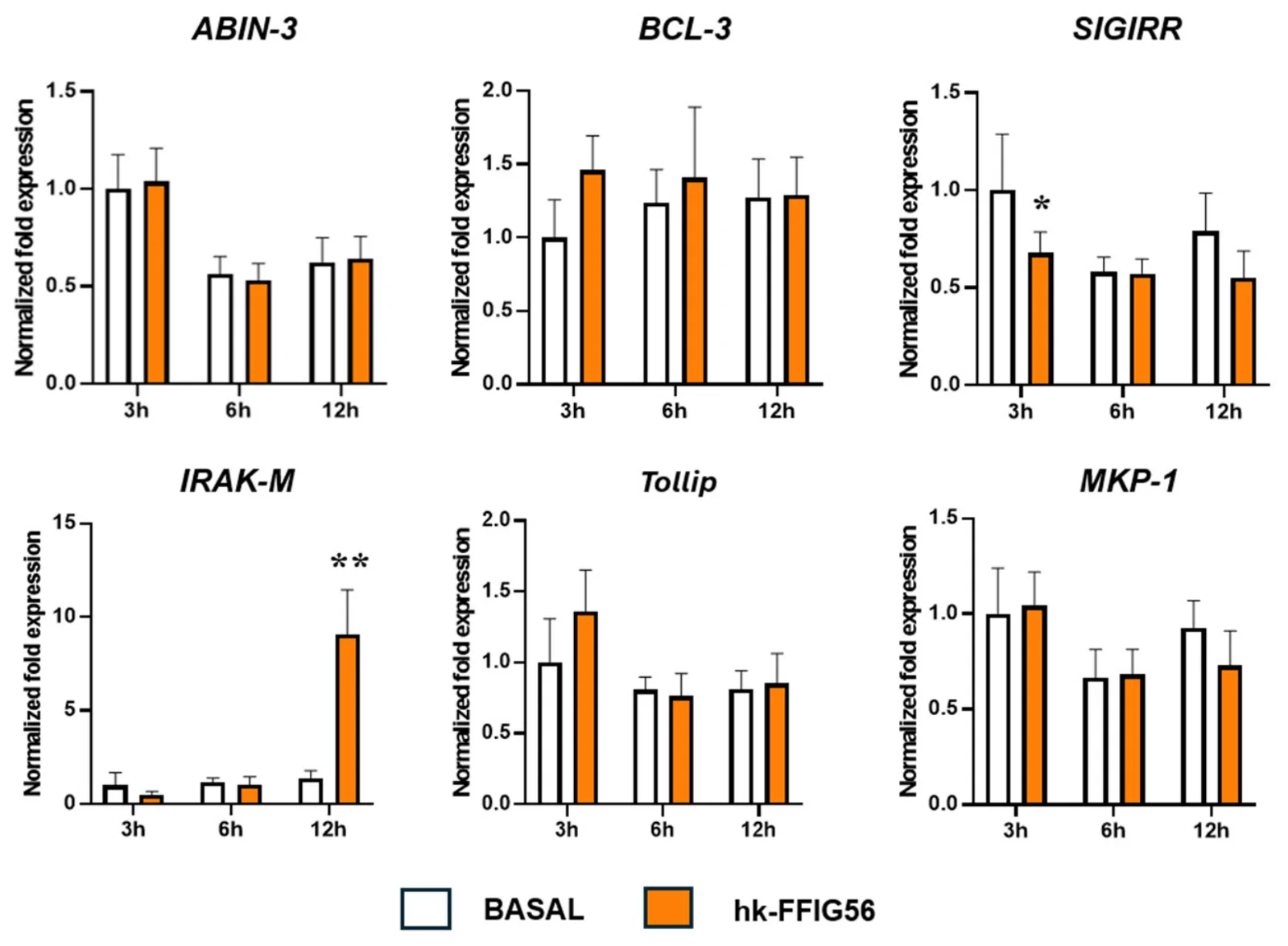
Effect of heat-killed *Ligilactobacillus salivarius* FFIG56 (hk-FFIG56) on the expression of negative regulators of inflammatory signaling in bovine mammary epithelial cells (BMECs). BMECs were stimulated with hk-FFIG56 in the absence of LPS challenge. The expression levels of *SIGIRR*, *Tollip*, *IRAK-M*, *ABIN-3*, *MKP-1*, and *BCL-3* were determined by RT-qPCR at 3, 6, and 12 h after hk-FFIG56 stimulation. Untreated BMECs were used for comparison. The results are shown as mean ± SD (*n* = 3 biological replicates per experimental condition). Significant differences are shown compared to the non-stimulated control group at the same time point, *p* < 0.05 (*), *p* < 0.01 (**).

We have previously reported that immunobiotic bacteria can modulate the response of BMECs to inflammatory stimuli by modifying the expression of negative regulators of TLR signaling [17]. In order to find out whether the postimmunobiotic hk-FFIG56 exerted a similar effect, we evaluated the expression of the negative regulators: single Ig IL-1-related receptor (*SIGIRR*), Toll-interacting protein (*Tollip*), interleukin-1 receptor associated kinase-M (*IRAK-M*), A20-binding inhibitor of NF-κB activation 3 (*ABIN-3*), mitogen-activated protein kinase phosphatase 1 (*MKP-1*), and B-cell 3 (*BCL-3*). The first set of experiments was performed in the absence of LPS stimulation (Figure 4). No differences were detected between control and hk-FFIG56-treated BMECs when the expression of *ABIN-3*, *BCL-3*, *Tollip* and *MKP-1* was evaluated after 3, 6 or 12 h. In contrast, it was observed that hk-FFIG56 significantly reduced *SIGIRR* expression at hour 3 and increased *IRAK-M* expression at hour 12 (Figure 4).

**Figure 5:**
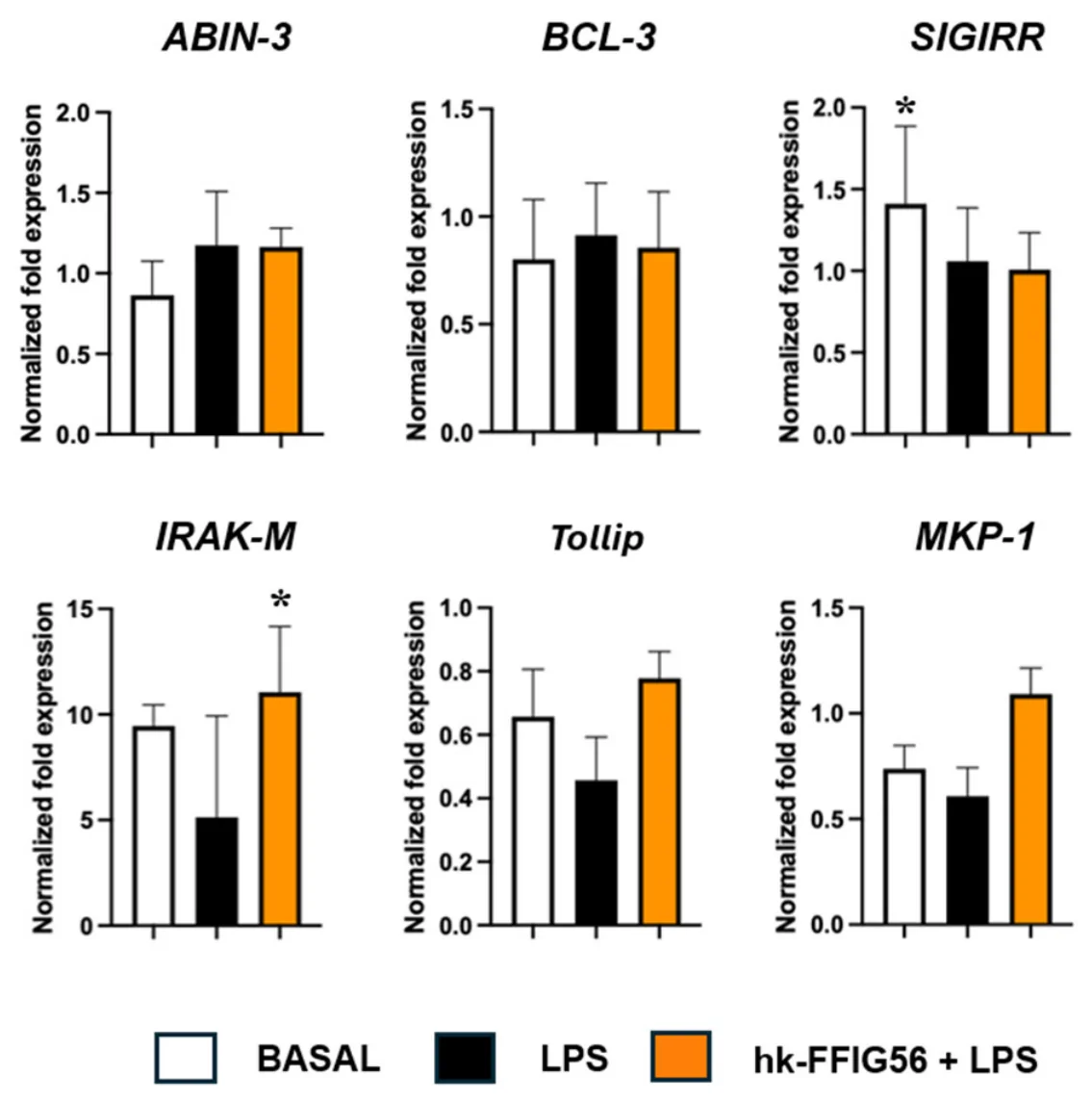
Effect of heat-killed *Ligilactobacillus salivarius* FFIG56 (hk-FFIG56) on the expression of negative regulators of inflammatory signaling in LPS-challenged bovine mammary epithelial cells (BMECs). BMECs were pre-stimulated with hk-FFIG56 and subsequently challenged with LPS. The expression levels of *SIGIRR*, *Tollip*, *IRAK-M*, *ABIN-3*, *MKP-1*, and *BCL-3* were determined by RT-qPCR at 3, 6, and 12 h after LPS stimulation. BMECs stimulated with LPS without hk-FFIG56 pretreatment were used for comparison. The results are shown as mean ± SD (*n* = 3 biological replicates per experimental condition). Significant differences are shown compared to the LPS-stimulated control group at the same time point, *p* < 0.05 (*).

In the second set of experiments, both control and hk-FFIG56-treated BMECs were stimulated with LPS, and the expression of negative regulators was evaluated after 3, 6 or 12 h (Figure 5). The challenge with LPS did not change the expression of *ABIN-3*, *BCL-3*, *IRAK-M* or *MKP-1*, while it decreased *SIGIRR* and *Tollip*. On the other hand, BMECs treated with hk-FFIG56 had higher levels of *IRAK-M*, *Tollip* and *MKP-1* than LPS-challenged control cells (Figure 5).

### 2.4. Modulation of NF-κB Signaling Pathway in BMECs by hk-FFIG56

**Figure 6:**
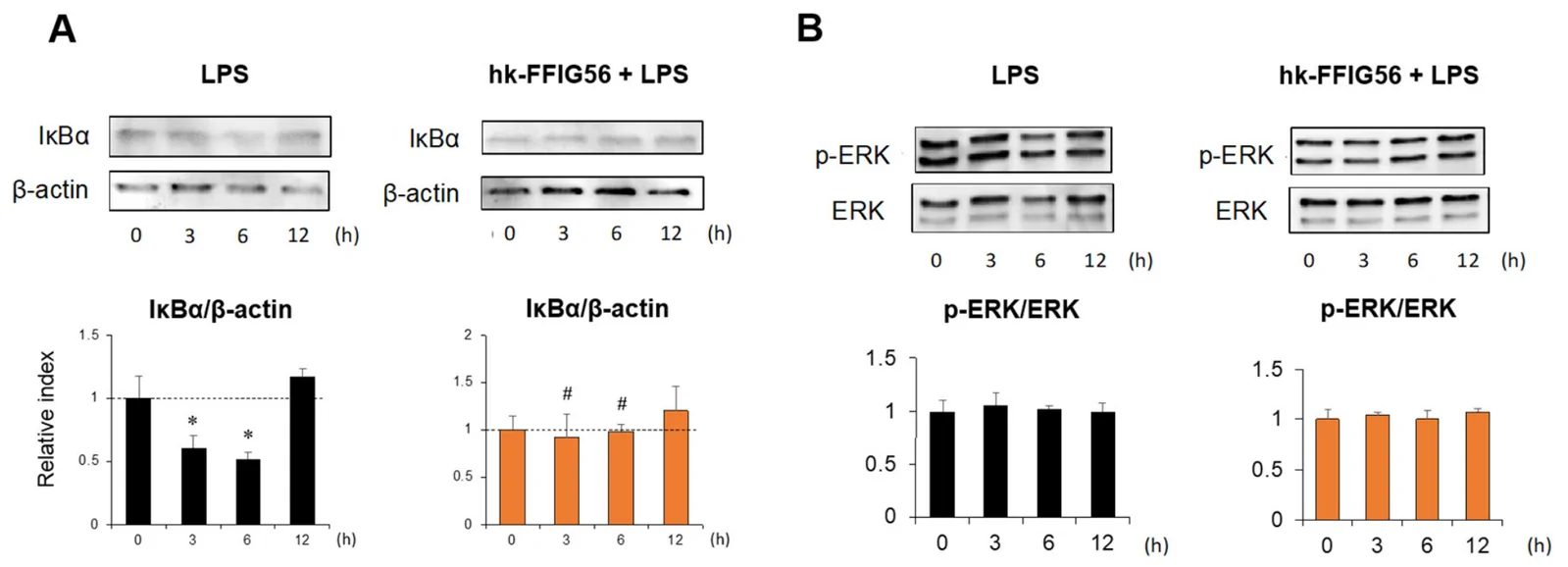
Effect of heat-killed *Ligilactobacillus salivarius* FFIG56 (hk-FFIG56) on NF-κB and MAPK-ERK signaling pathways in LPS-challenged bovine mammary epithelial cells (BMECs). BMECs were pre-stimulated with hk-FFIG56 and subsequently challenged with LPS. The activation of the NF-κB and MAPK-ERK signaling pathways was evaluated by Western blot at 3, 6, and 12 h after LPS stimulation. (**A**) IκBα protein levels and IκBα/β-actin ratios were evaluated as indicators of NF-κB pathway activation. (**B**) Phosphorylated ERK (p-ERK) and total ERK protein levels and p-ERK/ERK ratios were evaluated as indicators of MAPK-ERK pathway activation. BMECs stimulated with LPS without hk-FFIG56 pretreatment were used for comparison. The results are shown as mean ± SD (*n* = 3 biological replicates per experimental condition). Statistical differences were evaluated using two-way ANOVA, with treatment and time as independent factors, followed by multiple-comparison testing. Significant differences relative to time 0 within each treatment group are indicated by asterisks (*, *p* < 0.05). Significant differences between LPS-stimulated BMECs with and without hk-FFIG56 pretreatment at the corresponding time points are indicated by hash symbols (#, *p* < 0.05).

Considering the ability of hk-FFIG56 to modulate negative regulators of TLRs in BMECs, we next aimed to evaluate whether these changes translated into modulation of the key signaling pathways that control the expression of inflammatory factors in BMECs. Then, selected indicators of the NF-κB (nuclear factor kappa-B) and MAPK-ERK (mitogen-activated protein kinase/extracellular signal-regulated kinase) pathways were evaluated by Western blot (Figure 6). It was observed that LPS stimulation induced a decrease in the IκBα/β-actin ratio in control BMECs at hours 3 and 6, consistent with activation of NF-κB signaling. In contrast, hk-FFIG56 pretreatment attenuated the LPS-induced reduction in the IκBα/β-actin ratio, with significantly higher values observed in hk-FFIG56-pretreated BMECs than in cells stimulated with LPS alone at the corresponding time points (Figure 6A). On the other hand, no modifications of the p-ERK/ERK ratios were detected in both control and hk-FFIG56-treated BMECs (Figure 6B).

### 2.5. Modulation of κ-Casein Expression in BMECs by hk-FFIG56

**Figure 7:**
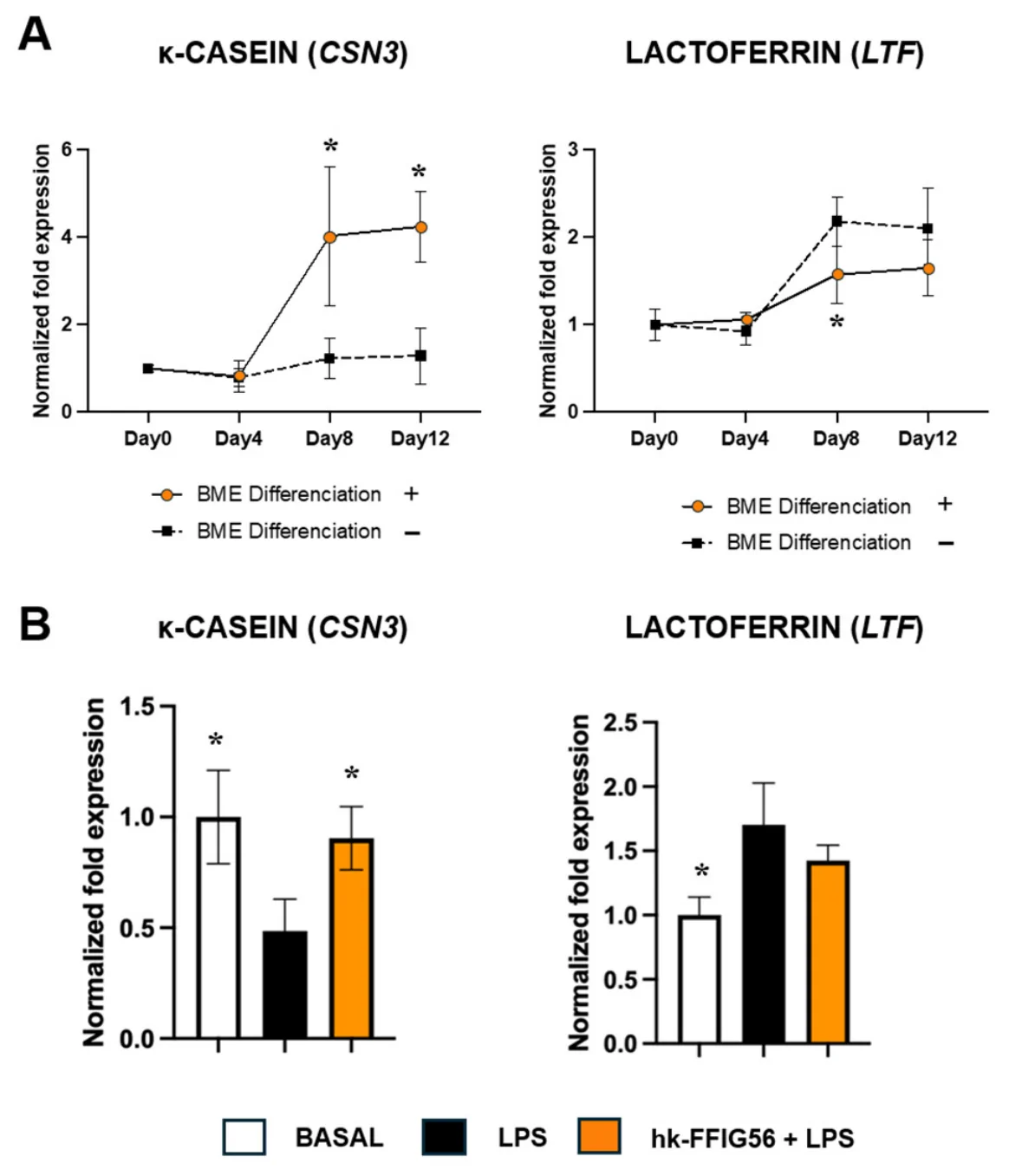
Effect of heat-killed *Ligilactobacillus salivarius* FFIG56 (hk-FFIG56) and LPS stimulation on the mRNA expression of κ-casein (*CSN3*) and lactoferrin (*LTF*) in bovine mammary epithelial cells (BMECs). (**A**) BMECs were cultured in standard or differentiation medium, and the expression levels of κ-casein and lactoferrin genes were evaluated at different time points. The results are shown as mean ± SD (*n* = 3 biological replicates per experimental condition). Significant differences are shown compared to the control group growing in no-differentiation medium at the same time point, *p* < 0.05 (*). (**B**) BMECs were cultured in differentiation medium for 7 days, stimulated with hk-FFIG56 for 12 h, and subsequently challenged with LPS for an additional 12 h. The expression levels of κ-casein and lactoferrin genes were then evaluated. BMECs treated with LPS and/or hk-FFIG56 were compared with untreated control cells. The results are shown as mean ± SD. Significant differences are shown compared to the LPS-stimulated control group, *p* < 0.05 (*).

Finally, we aimed to assess whether the treatment of BMECs with LPS and/or hk-FFIG56 modified the mRNA expression of the milk protein-related genes encoding κ-casein (*CSN3*) and lactoferrin (*LTF*). First, we evaluated the changes in the expression of these two genes in BMECs treated with a differentiation medium. As shown in Figure 7A, BMECs growing in the differentiation medium expressed significantly higher levels of *CSN3* from day 8 to 12 compared to cells growing in the standard medium. The expression of *LTF* increased on days 8 and 12 of culture in BMECs growing in both differentiation and standard mediums; however, the levels of this gene were significantly lower in the differentiation medium on day 8 (Figure 7A). Considering these results, we selected the differentiation medium and day 8 for further experiments.

As described in the Materials and Methods Section, BMECs were grown in the differentiation medium for 7 days, treated with hk-FFIG56 for 12 h and stimulated with LPS for an additional 12 h, and the expression of the *CSN3* and *LTF* genes was evaluated (Figure 7B). Hk-FFIG56 significantly increased *CSN3*, while no effect was observed for *LTF* compared to LPS-challenged control BMECs.

## 3. Discussion

Mammary epithelial cells play a central role in mammary gland physiology by producing milk components and serving as the first line of defense against invading pathogens through the production of cytokines, chemokines, interferons, and antimicrobial peptides that orchestrate innate immune responses and promote pathogen clearance. Given their central role in both milk production and host defense, understanding how mammary epithelial cells regulate innate immune signaling pathways is essential for elucidating the mechanisms underlying host–pathogen interactions during mastitis. Consistent with the central role of mammary epithelial cells in innate immune responses, the results of the present study showed that LPS stimulation markedly increased the expression of the cytokine *IL-6* and the chemokines *IL-8*, *CCL2*, *CXCL2*, and *CXCL3* in BMECs at 6 h post-stimulation, with elevated levels maintained up to 12 h. These findings are in agreement with our previous observations in LPS-stimulated BMECs, where the expression of inflammatory factors was significantly upregulated at 6 h and remained elevated for up to 24 h [16].

Given the prominent role of these mediators in the recruitment and activation of immune cells during mastitis, modulation of their expression represents an important mechanism through which immunomodulatory bacteria may regulate inflammatory responses in the mammary epithelium. In this context, our results demonstrate that pretreatment of BMECs with hk-FFIG56 significantly altered the LPS-induced transcriptional response, suggesting that this postimmunobiotic treatment can modulate epithelial innate immune signaling. Several studies have demonstrated the capacity of probiotic LAB to modulate epithelial and immune responses during bovine mastitis, most of them using viable bacteria. In vitro experiments on bovine mammary epithelial cells (MAC-T cells) showed that live *Lacticaseibacillus rhamnosus* GR-1 protects against *E. coli*-induced apoptosis by activating PINK1/Parkin-mediated mitophagy, thereby preserving mitochondrial integrity while attenuating inflammatory signaling and the expression of pro-inflammatory mediators [18]. Similarly, using both in vitro experiments in bovine mammary epithelial cells and an in vivo mouse model of mastitis, it was demonstrated that the live strain *Lacticaseibacillus casei* 03 exerts protective effects against *E. coli*-induced mastitis by modulating host immune responses, attenuating TLR4-mediated inflammatory signaling, and reducing the production of pro-inflammatory cytokines, including TNF-α, IL-1β, and IL-6 [19]. In addition, it was reported that viable *Lactiplantibacillus plantarum* 17-5 attenuates *E. coli*-induced inflammatory responses in primary cultures of bovine mammary epithelial cells by reducing *TNF-α*, *IL-1β*, and *IL-6* expression through inhibition of the NF-κB and MAPK signaling pathways [20].

In addition to these studies using live bacteria, it was demonstrated in an in vitro mastitis model based on MAC-T cells that heat-inactivated *Lactobacillus gasseri* LA806 partially preserved the barrier-protective and immunomodulatory effects of the live strain, regulating the expression of inflammatory mediators and cytokines and suggesting that non-viable probiotic preparations can retain significant immunomodulatory activity [21]. Our results are consistent with this previous work, as we demonstrated that the non-viable bacterium FFIG56 is capable of differentially modulating the expression of immunological factors in BMECs in response to TLR4 signaling activation. Our data showed that hk-FFIG56 reduced the expression of *IL-6*, *IL-8*, and *CCL2* and prevented the LPS-induced reduction in IκBα protein levels, suggesting an effect on NF-κB-associated signaling. In contrast, no significant changes in the p-ERK/ERK ratio were detected at the evaluated time points. Furthermore, the pretreatment with hk-FFIG56 increased IRAK-M, Tollip and MKP-1 expression levels after LPS stimulation, suggesting that this postimmunobiotic may regulate inflammatory responses through the modulation of endogenous regulatory mechanisms in BMECs. In addition, the lack of significant changes in ERK phosphorylation despite the changes in the expression levels of MKP-1 suggests that the modulatory effects of hk-FFIG56 are more closely associated with NF-κB than with the MAPK-ERK pathway. In contrast, our previous study using BMECs pretreated with *Lactobacillus acidophilus* CRL2074 demonstrated an upregulation of SIGIRR and ABIN-3 following LPS challenge [17]. These differences indicate that immunobiotic-/postimmunobiotic-mediated regulation of negative feedback mechanisms within TLR signaling pathways is highly strain-dependent, highlighting distinct immunomodulatory strategies among probiotic strains.

In bovine mammary epithelial cells, LPS stimulation has been shown to induce a robust inflammatory response mediated by activation of the NF-κB signaling pathway. It was shown that LPS promotes the phosphorylation and degradation of IκBα, leading to the nuclear translocation and activation of NF-κB, which in turn drives the transcription of pro-inflammatory mediators such as *IL-6*, *IL-1β*, and *TNF-α*. Interestingly, the overexpression of Syndecan-3 attenuated NF-κB activation by stabilizing IκBα levels, resulting in a significant reduction in cytokine expression [22]. The treatment of MAC-T cells with allicin, a biologically active sulfur-containing compound produced from garlic, significantly attenuated their response to LPS by reducing the phosphorylation of IκB-α and NF-κB p65, thereby suppressing NF-κB activation and decreasing cytokine production [23]. These findings highlight that modulation of the TLR4/NF-κB axis directly impacts the expression of inflammatory mediators and represents a key mechanism for controlling inflammatory responses in the bovine mammary epithelium. Thus, it is possible to speculate that hk-FFIG56 could interact with PRRs expressed in BMECs, inducing changes in the expression of negative regulators, which would subsequently regulate TLR4/NF-κB axis activation and, consequently, the expression of inflammatory factors. Deciphering which molecule(s) present in hk-FFIG56 and which PRR(s) in BME mediate this bacteria–host interaction is an important point for future research. Functional studies involving the inhibition or silencing of specific negative regulators, together with additional analyses of intracellular signaling, will be necessary to determine whether these molecules directly mediate the anti-inflammatory effects of hk-FFIG56.

Interestingly, hk-FFIG56 pretreatment differentially modulated the expression of inflammatory mediators in LPS-challenged BMECs. While significant reductions were observed in *IL-6*, *IL-8*, and *CCL2* expression, no significant changes were detected in *CXCL2* or *CXCL3* expression. These findings suggest that the immunomodulatory activity of hk-FFIG56 does not uniformly affect all LPS-induced inflammatory genes but may involve selective regulation of specific inflammatory responses. Such differences could be associated with distinct transcriptional regulatory mechanisms or variations in the kinetics of gene expression following LPS stimulation. Therefore, the absence of significant changes in *CXCL2* and *CXCL3* under the experimental conditions evaluated does not exclude the possibility of modulation at other time points. Further studies investigating the temporal dynamics and regulatory mechanisms of these inflammatory mediators would help clarify the selective effects of hk-FFIG56 on mammary epithelial immune responses. An additional limitation of this study is that the effects of hk-FFIG56 alone on the inflammatory mediators evaluated in this work were not assessed. Therefore, although hk-FFIG56 pretreatment modified the subsequent response of BMECs to LPS stimulation, the present experiments do not allow us to distinguish these effects from potential changes in basal inflammatory gene expression induced by the bacterial preparation itself. Further studies including hk-FFIG56-only controls will be necessary to clarify this aspect.

Importantly, the reduction in inflammatory mediator expression observed following hk-FFIG56 pretreatment should not necessarily be interpreted as a beneficial effect on mammary host defense. *IL-8* and *CCL2* contribute to the recruitment of immune cells during infection, and their expression is essential for the establishment of an effective inflammatory response. While excessive or prolonged inflammation may contribute to mammary tissue damage, an insufficient inflammatory response could impair immune cell recruitment and pathogen clearance. In this context, previous studies reporting increased local inflammatory responses following intramammary administration of certain LAB strains are not necessarily contradictory to our findings, as the consequences of immune modulation depend on the bacterial strain, experimental setting, and stage of infection. In particular, the increased *IL-8* levels reported following intramammary administration of *L. lactis* DPC3147 were associated with local immune activation in infected animals [12], whereas our study evaluated the transcriptional response of isolated BMECs to LPS stimulation in the absence of viable pathogens and recruited immune cells. Therefore, although hk-FFIG56 pretreatment reduced the expression of selected inflammatory mediators, the present model cannot determine whether these changes would limit excessive inflammation or compromise protective immune responses during mammary infection. Further studies using infection models that allow the evaluation of immune cell recruitment, pathogen clearance, and tissue responses will be necessary to establish the physiological significance of these findings.

Bovine mammary epithelial cells are the primary functional cells of the mammary gland, responsible for the synthesis and secretion of milk components through tightly regulated biosynthetic pathways. Caseins constitute approximately 80% of total milk proteins, and κ-casein, located on the surface of casein micelles, plays a critical role in their stabilization and is essential for dairy product manufacturing. In contrast, lactoferrin is a whey protein that contributes to mammary gland defense by exerting antimicrobial and immunomodulatory effects [24]. During mammary gland infection, casein levels typically decrease, whereas lactoferrin and immunoglobulin concentrations increase, reflecting a shift from milk production to an inflammatory state [25]. In line with these observations, our results showed that LPS stimulation reduced *CSN3 (*κ-casein) mRNA levels while increasing *LTF* (lactoferrin) expression in BMECs. Notably, pretreatment with hk-FFIG56 attenuated the LPS-induced changes in the expression of *CSN3*. These findings suggest a potential effect on the transcriptional regulation of milk protein-related genes under inflammatory conditions. However, these transcriptional changes do not necessarily reflect alterations in protein abundance, milk quality, or mammary secretory function. Further studies evaluating protein levels and functional indicators of milk synthesis will be necessary to determine the physiological relevance of these findings. In addition, further studies are warranted to elucidate the underlying mechanisms of the beneficial effects of hk-FFIG56 in the regulation of milk protein synthesis. In particular, the effect on transcription factors associated with the JAK2–STAT5 [26], HIF-1α and β-catenin [27] signaling pathways should be evaluated considering the important role of these pathways in milk protein synthesis in bovine mammary epithelial cells.

## 4. Materials and Methods

### 4.1. Lactic Acid Bacteria

A total of forty-six strains of LAB belonging to the Culture Collection of the Laboratory of Animal Food Function, Graduate School of Agricultural Science, Tohoku University (Sendai, Japan), were used (Supplementary Table S2). LAB were cultivated in Man, Rogosa, and Sharpe broth (MRS; Difco, Detroit, MI, USA) at 37 °C for 16 h and washed with phosphate-buffered saline (PBS). After washing, the cells were heat-killed at 70 °C for 90 min. The heat-killed strains were then resuspended in PBS to a concentration of 2.5 × 10^9^ cells/mL and stored at −20 °C until testing.

### 4.2. Bovine Mammary Epithelial Cell Line

The bovine mammary epithelial cell (BMEC) line used in this study was previously established from mammary gland tissue obtained from a Holstein cow at 200 days of gestation and characterized for its responsiveness to mitogenic and lactogenic stimuli [16,28]. Cells were maintained following the culture protocol previously reported, and passages 22–28 were used for the experiments [16,28].

The immunomodulatory capacities of heat-killed LAB strains were examined using BMECs as described previously [16,28]. BMECs were grown in Dulbecco’s Modified Eagle Medium (DMEM; Thermo Fisher Scientific, Waltham, MA, USA) supplemented with 20% fetal calf serum (FCS; Sigma-Aldrich, St. Louis, MO, USA), 100 U/mL penicillin and 100 μg/mL streptomycin (Thermo Fisher Scientific), transferrin (10 ng/mL) (Sigma-Aldrich, MO, USA), and sodium acetate (5 mM) (FUJIFILM Wako Pure Chemical Corporation, Osaka, Japan). For the passage, BMECs were seeded at 1 × 10^4^/cm^2^ in a collagen-coated flask (Sumitomo Bakelite Co., Ltd., Tokyo, Japan) and incubated at 37 °C with 5% CO_2_. The cell culture medium was changed every 48 h, and three consecutive passages were conducted before the challenge experiment.

### 4.3. Study of LAB in BMECs

BMECs were seeded at 1.25 × 10^5^ cells/well in a 24-well plate and cultured for 2 days at 37 °C with 5% CO_2_. After reaching confluence (2.5 × 10^5^ cells/well), the heat-killed LAB strains (2.5 × 10^7^ cells/well) were added to the BMECs and cultured for 12 h at 37 °C with 5% CO_2_. Subsequently, the cells were washed twice with PBS and then stimulated with LPS (1.0 µg/mL, *Escherichia coli* O55, L6529, Sigma-Aldrich, Tokyo, Japan). This concentration was selected based on our previously established experimental model of LPS-induced inflammatory responses in BMECs [16,28]. BMECs were cultured for the specified times at 37 °C with 5% CO_2_. RNA-seq analysis was performed using samples collected 12 h after LPS stimulation. For RT-qPCR and Western blot analyses, samples were collected at 3, 6, and 12 h after LPS stimulation to evaluate temporal changes in inflammatory gene expression and selected signaling markers.

For the analysis of *CSN3* and *LTF* mRNA expression, BMECs were cultured in differentiation medium for 7 days before hk-FFIG56 pretreatment for 12 h and subsequent LPS stimulation for an additional 12 h.

### 4.4. RNA-Sequencing

Total RNA was extracted from the treated and untreated control cells (*n* = 3) using the PureLink RNA Mini Kit (Thermo Fisher Scientific, MA, USA). RNA integrity, quality, and quantity were evaluated with a microcapillary electrophoresis 2100 Bioanalyzer (Agilent Technologies, Santa Clara, CA, USA) using the Agilent RNA 6000 Nano Kit (Agilent Technologies, Santa Clara, CA, USA). Samples with an RNA integrity number (RIN) greater than 9.5 were used for RNA-sequence analysis. Each RNA sample was sent to Novogene Co., Ltd. (Tokyo, Japan) for the construction of Illumina mRNA libraries and subsequent sequencing. Libraries were generated using the NEB Next^®^ Ultra™ Directional RNA Library Prep Kit (NEB, Ipswich, MA, USA) following the manufacturer’s protocol. The library was checked with Qubit and real-time PCR for quantification and Bioanalyzer for size distribution detection. Quantified libraries were pooled and sequenced on Illumina platforms according to effective library concentration and data amount. The clustering of the index-coded samples was performed according to the manufacturer’s instructions. After cluster generation, the library preparations were sequenced on an Illumina platform and paired-end reads were generated.

### 4.5. RNA-Sequencing Data Analysis

Raw sequencing reads were subjected to quality control using FastQC (v11.9) to assess sequence quality. Adapters and low-quality bases were trimmed using prinseq-lite v.0.20.4. The trimmed reads were then aligned to the reference genome (*Bos taurus* ARS-UCD1.2) using STAR (v2.7.10). Gene-level expression quantification was performed using featureCounts (v2.0.5) with the Ensembl *Bos taurus* ARS-UCD1.2 gene annotation as a reference. The count matrix obtained from featureCounts was imported into R (v4.3.2) for downstream analysis using the DESeq2 package (v1.42.0). Differential expression analysis was performed using DESeq2. Genes with an adjusted *p*-value < 0.05 and absolute log_2_ fold change > 1 (|log_2_FC| > 1) were considered differentially expressed. Functional annotation and enrichment analysis were conducted using the PANTHER Classification System (v17.0). Gene Ontology (GO) terms were assigned to elucidate the biological functions, cellular components, and molecular functions of the annotated genes.

### 4.6. Quantitative Real-Time PCR

Total RNA was extracted from BMECs according to our previous publication [16,28]. Briefly, total RNA was collected and purified using TRIzol reagent (Molecular Research Center, OH, USA) following the manufacturer’s protocol. The concentration of total RNA was measured by NanoDrop™ One (Thermo Fisher Scientific, MA, USA). cDNA synthesis was performed using the PrimeScript RT Reagent Kit (Takara Bio, Shiga, Japan) following the manufacturer’s recommendations. RT-qPCR was conducted using the CFX Connect real-time PCR system (Bio-Rad, Los Angeles, CA, USA) with Platinum SYBR Green qPCR Super Mix—UDG with ROX (Thermo Fisher Scientific, MA, USA) to quantify the mRNA of immune-related factors in BMECs. The thermal cycling conditions for RT-qPCR were 2 min at 50 °C, followed by 5 min at 95 °C, then 40 cycles of 15 s at 95 °C, 30 s at 60 °C, and 30 s at 72 °C. The primers used in this study were described previously [16,28] (Supplementary Table S3). *β-actin* was used as a housekeeping gene, and the mRNA expression levels of each gene were calculated using a standard curve obtained by serial dilution of plasmid DNA and expressed as normalized fold expression relative to the mRNA expression of *β-actin*.

### 4.7. Western Blotting

BMECs were stimulated with heat-killed LAB (5.0 × 10^7^ cells/mL) for 12 h. Cells were then stimulated with LPS (1.0 μg/mL) and incubated for an additional 3, 6, or 12 h at 37 °C under 5% CO_2_. After stimulation, cells were harvested using the EzRIPA Lysis Kit (ATTO, Tokyo, Japan), lysed, and centrifuged at 15,000 rpm for 10 min at 4 °C. The resulting supernatants were collected, and protein concentrations were determined using a BCA protein assay kit (Thermo Fisher Scientific, MA, USA) and normalized across samples. Sample buffer containing 2-mercaptoethanol (2ME+) (FUJIFILM Wako Pure Chemical, Osaka, Japan) was added to the protein samples, which were then heat-denatured at 95 °C for 5 min. Proteins were separated by SDS-PAGE using 10% Mini-PROTEAN TGX gels (Bio-Rad, CA, USA) at 125 V for 60 min and subsequently transferred onto PVDF membranes using the Trans-Blot Turbo RTA Transfer Kit (Bio-Rad) at 25 V and 2.5 A for 7 min. Membranes were washed five times with TBS-T (FUJIFILM Wako Pure Chemical, Japan) and blocked with EveryBlot Blocking Buffer (Bio-Rad). Membranes were incubated overnight at 4 °C with gentle agitation using primary antibodies against β-actin (1:1000; #4970, Cell Signaling Technology, Danvers, MA, USA), IκBα (1:1000; #9242S, Cell Signaling Technology, MA, USA), total ERK1/2 (1:1000; #9102, Cell Signaling Technology, MA, USA), and phosphorylated ERK1/2 (1:500; Phospho-p44/42 MAPK (Thr202/Tyr204), #9101 Cell Signaling Technology, MA, USA). After four washes with TBS-T, membranes were incubated for 1 h at room temperature with an alkaline phosphatase-conjugated goat anti-rabbit IgG secondary antibody (1:2000; Proteintech, Rosemont, IL, USA). Following extensive washing, immunoreactive bands were visualized using ECF substrate (GE Healthcare, Chicago, IL, USA) and detected with a ChemiDoc MP Imaging System (Bio-Rad). Band intensities were quantified using the Image Lab software ver. 6.1 (Bio-Rad). The relative intensities of the immunoreactive bands were determined by densitometric analysis using the Image Lab software (Bio-Rad). IκBα protein levels were normalized to the corresponding β-actin band intensities, whereas ERK phosphorylation was expressed as the ratio of phosphorylated ERK to total ERK (p-ERK/ERK).

### 4.8. Differentiation of BMECs

BMECs were grown at a concentration of 2.7 × 10^5^ cells/well in collagen type IV-hcoated 24-well plates (Corning, Corning, NY, USA) and cultured for 1 day at 37 °C, 5% CO_2_. Subsequently, cells were cultured in differentiation-inducing medium DMEM:F12—which contained 5% fetal calf serum, prolactin (2 µg/mL), 5 µg/mL insulin (Sigma-Aldrich, St. Louis, MO, USA), 100 nM dexamethasone (Sigma-Aldrich, St. Louis, MO, USA), 5 µg/mL growth hormone (Protein Laboratories Rehovot Ltd., Rehovot, Israel), epidermal growth factor (10 ng/mL), penicillin (100 U/mL), and streptomycin (100 µg/mL)—for 12 days, maintaining the temperature at 37 °C with 5% CO_2_. The differentiation-inducing medium was changed every two days. After 7 days of differentiation induction treatment, heat-killed LAB (2.5 × 10^7^ cells/well) were applied for 12 h, followed by two washes with PBS, and then LPS was applied for 12 h.

### 4.9. Statistical Analysis

Statistical analyses were performed using GraphPad Prism 10 (GraphPad Software, CA, USA). Experiments were performed using three independent biological replicates, each including two technical replicates per experimental condition. Technical replicates were averaged within each independent experiment, and biological replicates were considered the experimental units for statistical analysis (*n* = 3). Data are presented as mean ± standard deviation (SD). For experiments evaluating the effects of hk-FFIG56 pretreatment and LPS stimulation at different time points, two-way ANOVA was used, with treatment condition and time after LPS stimulation as the independent factors, followed by Tukey’s multiple comparisons test to evaluate differences between treatment groups at the corresponding time points, particularly between LPS-stimulated cells with and without hk-FFIG56 pretreatment. For the differentiation experiments, two-way ANOVA followed by Tukey’s multiple comparisons test was performed using culture medium and differentiation time as the independent factors. For experiments comparing multiple treatment groups at a single time point, one-way ANOVA followed by Tukey’s multiple comparisons test was used. For RNA-seq analysis, differential gene expression was evaluated using DESeq2, with Benjamini–Hochberg-adjusted *p*-values used to control the false discovery rate, as described in the RNA-seq analysis section. Statistical significance was defined as *p* < 0.05.

## 5. Conclusions

In conclusion, this study demonstrated that heat-killed *Ligilactobacillus salivarius* FFIG56 (hk-FFIG56) modulates LPS-induced inflammatory responses in bovine mammary epithelial cells. These effects were associated with increased expression of negative regulators of TLR signaling and changes in IκBα protein levels, suggesting a potential involvement of NF-κB-associated regulatory mechanisms. Furthermore, hk-FFIG56 pretreatment attenuated an LPS-induced reduction in *CSN3* mRNA expression in differentiated bovine mammary epithelial cells, although its effects on κ-casein protein abundance and milk production remain to be determined. Together, these findings provide evidence of the immunomodulatory activity of hk-FFIG56 under the experimental conditions evaluated and support further investigation of its biological properties. Additional mechanistic studies and in vivo evaluations are required to determine whether these effects can be translated into beneficial outcomes for bovine mammary health.

## Data Availability

The data presented in this study are available throughout the article.

## Supplementary Materials

The following supporting information can be downloaded at https://www.mdpi.com/article/10.3390/ijms27219809/s1.

## Abbreviations

The following abbreviations are used in this manuscript:

BCA	Bicinchoninic acid
BCL-3	B-cell lymphoma 3
BME	Bovine mammary epithelial
CCL2	C-C motif chemokine ligand 2
CSN3	κ-casein
CXCL2	C-X-C motif chemokine ligand 2
CXCL3	C-X-C motif chemokine ligand 3
CXCL8	C-X-C motif chemokine ligand 8
DMEM	Dulbecco’s Modified Eagle Medium
ECF	Enhanced chemifluorescence
ERK	Extracellular signal-regulated kinase
FCS	Fetal calf serum
HIF-1α	Hypoxia-inducible factor 1 alpha
hk-FFIG56	Heat-killed *Ligilactobacillus salivarius* FFIG56
hk-LAB	Heat-killed lactic acid bacteria
IL	Interleukin
IRAK-M	Interleukin-1 receptor-associated kinase M
JAK2	Janus kinase 2
LAB	Lactic acid bacteria
LPS	Lipopolysaccharide
LTF	Lactoferrin
MAMP	Microbe-associated molecular pattern
MAPK	Mitogen-activated protein kinase
MCP-1	Monocyte chemoattractant protein 1
MKP-1	Mitogen-activated protein kinase phosphatase 1
MRS	Man, Rogosa, and Sharpe
NF-κB	Nuclear factor kappa B
PBS	Phosphate-buffered saline
PCR	Polymerase chain reaction
PRR	Pattern recognition receptor
qPCR	Quantitative polymerase chain reaction
RT-qPCR	Reverse transcription quantitative polymerase chain reaction
SDS-PAGE	Sodium dodecyl sulfate–polyacrylamide gel electrophoresis
SIGIRR	Single Ig IL-1-related receptor
STAR	Spliced Transcripts Alignment to a Reference
STAT5	Signal transducer and activator of transcription 5
TBS-T	Tris-buffered saline with Tween
TLR	Toll-like receptor
TLR4	Toll-like receptor 4
TNF-α	Tumor necrosis factor alpha
Tollip	Toll-interacting protein
TRIzol	Tri-reagent solution
