# Supplementary material for: Heat-Killed Ligilactobacillus salivarius FFIG56 Modulates Innate Immune Response in Bovine Mammary Epithelial Cells
Source: Int J Mol Sci. 2026 Nov 1;27(21):9809. doi: 10.3390/ijms27219809 (PMC13763847; doi:10.3390/ijms27219809)
Supplement: Supplementary file 1 [file ijms-27-09809-s001.zip › Supplementary Tables S2 and S3.pdf]

Supplementary Table S1. Transcriptomic profiles of bovine mammary epithelial (BME) cells following stimulation with heat-killed Ligilactobacillus salivarius FFIG56 (hk-FFIG56) and/or lipopolysaccharide (LPS). Gene expression data obtained by RNA sequencing are provided for BME cells stimulated with LPS, hk-FFIG56, or pre-stimulated with hk-FFIG56 and subsequently challenged with LPS (hk-FFIG56+LPS). Gene symbols, ensembl gene identifiers, expression values, and gene descriptions are provided.

Excel file is provided.

Supplementary Table S2. List of lactic acid bacteria strains used in this study.

Numbering	Strain	Species	Origin
1	LAFF7	*Lactiplantibacillus plantarum*	Vagina
2	LAFF8	*Lactiplantibacillus plantarum*	Vagina
3	LAFF9	*Lactiplantibacillus plantarum*	Vagina
4	LAFF13	*Lactiplantibacillus plantarum*	Colostrum
5	LAFF14	*Lactiplantibacillus plantarum*	Colostrum
6	LAFF15	*Lactiplantibacillus plantarum*	Colostrum
7	LAFF16	*Lactiplantibacillus plantarum*	Colostrum
8	LAFF17	*Lactiplantibacillus plantarum*	Colostrum
9	LAFF33	*Lactiplantibacillus plantarum*	Colostrum
10	LAFF34	*Lactiplantibacillus plantarum*	Colostrum
11	LAFF35	*Lactiplantibacillus plantarum*	Colostrum
12	LAFF36	*Lactiplantibacillus plantarum*	Colostrum
13	LAFF44	*Lactiplantibacillus plantarum*	Milk
14	LAFF45	*Lactiplantibacillus plantarum*	Milk
15	LAFF48	*Lactiplantibacillus plantarum*	Milk
16	LAFF49	*Lactiplantibacillus plantarum*	Milk
17	LAFF50	*Lactiplantibacillus plantarum*	Milk
18	LAFF98	*Lactiplantibacillus plantarum*	Vagina
19	LAFF100	*Lactiplantibacillus plantarum*	Vagina
20	LAFF138	*Lactiplantibacillus plantarum*	Vagina

21	LAFF149	*Lactiplantibacillus plantarum*	Colostrum
22	LAFF154	*Lactiplantibacillus plantarum*	Colostrum
23	LAFF155	*Lactiplantibacillus plantarum*	Colostrum
24	LAFF286	*Lactiplantibacillus plantarum*	Feces of piglet
25	LAFF291	*Lactiplantibacillus plantarum*	Feces of piglet
26	LAFF382	*Lactiplantibacillus plantarum*	Feces of piglet
27	LAFF411	*Lactiplantibacillus plantarum*	Feces of piglet
28	LAFF691	*Lactiplantibacillus plantarum*	Milk
29	LAFF692	*Lactiplantibacillus plantarum*	Milk
30	LAFF690	*Lactiplantibacillus plantarum*	Milk
31	LAFF694	*Lactiplantibacillus plantarum*	Vagina
32	LAFF695	*Lactiplantibacillus plantarum*	Vagina
33	LAFF700	*Lactiplantibacillus plantarum*	Vagina
34	LAFF701	*Lactiplantibacillus plantarum*	Vagina
35	LAFF704	*Lactiplantibacillus plantarum*	Milk
36	LAFF705	*Lactiplantibacillus plantarum*	Milk
37	LAFF706	*Lactiplantibacillus plantarum*	Milk
38	LAFF707	*Lactiplantibacillus plantarum*	Milk
39	LAFF797	*Lactiplantibacillus plantarum*	Feces of piglet
40	LAFF798	*Lactiplantibacillus plantarum*	Feces of piglet
41	LAFF805	*Lactiplantibacillus plantarum*	Feces of piglet
42	LAFF815	*Lactiplantibacillus plantarum*	Feces of piglet

43	LAFF816	*Lactiplantibacillus plantarum*	Feces of piglet
44	LAFF825	*Lactiplantibacillus plantarum*	Feces of piglet
45	FFIG56	*Ligilactobacillus salivarius*	Jejunum
46	FFIG67	*Ligilactobacillus salivarius*	Jejunum

Supplementary Table S3. Primer sequences used in this study

Gene symbol	Sequence (3` to 5`) *	Amplicon size
ACTB (β-actin)	F: TGG ATT GGC GGC TCC AT	57
	R: GCT GAT CCA CAT CTG CTG GAA	
CCL2/MCP-1	F: CAC CAG CAG CAA GTG TCC TAA A	65
	R: CAC ATA ACT CCT TGC CCA GGA T	
CXCL8/IL-8	F: TGC TCT CTT GGC AGC TTT CC	61
	R: TCT TGA CAG AAC TGC AGC TTC AC	
IL-6	F: CCA CCC CAG GCA GAC TAC TTC	63
	R: CCA TGC GCT TAA TGA GAG CTT	
CXCL2	F: CTA GGC CAG CTC TAA CTG AC	107
	R: TGG TGA TTC CTC TTT TCC CT	
CXCL3	F: GAC AGT TCC TGA AAA GTG GT	104
	R: ATA GTC CAG CAC ATC AAG TC	
ABIN-3	F: AAA GTG GGC TGC ATG TAC TTT GG	121
	R: AGG CTG TGG GAT GGC TTT C	
Tolllip	F: CGG GCG TGG ACT CTT TCT AC	65
	R: GAT GCG GTC GTC CAT GGA	
SIGIRR	F: GGC AGT GAA GTG GAT GTG TCA	56
	R: TCC GTG CGG GCA CTG TA	
Bcl-3	F: CAT GGA ACA CCC CCT GTC A	66
	R: GGC GTA TCT CCA TCC TCA TCA	
IRAK-M	F: CAG TAC TTG CAC AAC ACG GAA CTC	88
	R: TGG GTT GAA ACT GAT CAT CCA GAA	
MKP-1	F: CAG TTT GGG TTT GGC GTA AAG A	105
	R: AGC GTG CAA GTC GTC CTG TG	
CSN3 (κ-casein)	F: CAA CTG AAC CTA CTG CCA AGC AAG	97
	R: AGG GTT AAT GCC AGG ATA GTC ACA A	
LTF (Lactoferrin)	F: GAACCATACTTCGGTTATTCTGGTG	150
	R: CCGACTGTTGTTCAGGCAGAG	

*F: Forward, R: Reverse
